# Cellular Automata Modelling of Discontinuous Precipitation

**DOI:** 10.3390/ma14174985

**Published:** 2021-08-31

**Authors:** Jarosław Opara, Boris Straumal, Paweł Zięba

**Affiliations:** 1 Łukasiewicz Research Network—Institute for Ferrous Metallurgy, K. Miarki Str. 12, 44-100 Gliwice, Poland; 2 Institute of Solid State Physics of the Russian Academy of Sciences, 142432 Chernogolovka, Russia; straumal@issp.ac.ru; 3 Institute of Metallurgy and Materials Science of Polish Academy of Sciences, Reymonta Str. 25, 30-059 Cracow, Poland; p.zieba@imim.pl

**Keywords:** discontinuous precipitation, cellular automata, solute concentration profiles, modelling, digital material representation

## Abstract

The fundamentals of discontinuous precipitation (DP) reaction modelling using a cellular automata (CA) method are presented. In the proposed CA model, cell states, internal variables, equations, and transition rules were defined to predict the manner of mass transport during DP reaction and to relate changes in the microstructure with corresponding changes in chemical composition. Furthermore, the concept of digital material representation (DMR) was introduced into the CA model, which allowed schematic images of the microstructure to be used as starting structures in the modelling of the DP reaction. The preliminary assumptions adopted in the proposed CA model for the DP reaction were verified by numerical simulations of the growth of discontinuous precipitates at a steady-state at the example of Al-22 at.% Zn alloy. The outcomes achieved from the CA simulations were presented in a different form than that most commonly used previously (single concentration profiles), namely as the 2D maps showing changes in Zn content accompanying the successive stages of growth of discontinuous precipitates. The model used for the description of the solute diffusion along of the reaction front (RF) allowed two-dimensional systems at the nano-scale to be treated within a reasonable simulation time. The obtained results indicate that the developed CA model was able to realistically simulate the DP reaction, which was confirmed by the visualisation of migrating RFs together with associated chemical composition changes in the microstructure.

## 1. Introduction

Knowledge about microstructure and chemistry down to the nanometre level, which accompanies the formation and growth of new phase(s) during solid-solid state phase transformations, is crucial to developing a better understanding of their mechanisms and kinetics. This especially holds true if a two-phase lamellar product is formed. Here, discontinuous precipitation (DP) is a relevant example, as during this reaction not only does a new solute rich phase β form, but the whole process is limited to a moving reaction front (RF) within a high-angle grain boundary (GB). The process takes place according to a scheme: α_ο_ → α + β, which differs from the eutectoid transformation, where two new phases are formed (γ → α + β).

Bearing in mind the success in modelling of multi-physics phenomena in metallic materials at the grain scale, which was recently reviewed by Diehl [1], one can consider that modelling of the DP reaction in terms of the micro-scale, or even nano-scale, is also feasible. The quantitative modelling of DP is a challenging task because the process is extremely complex and controlled by a large number of parameters, which are often not precisely known. Furthermore, this issue requires operation at the nanometre level for the RF area discretisation, which is crucial in the model formulation and its efficiency. Nevertheless, some attempts to model the DP reaction were performed.

The first interesting result was obtained by Ramanarayan and Abinandanan [2]. They extended the phase field (PF) model, describing the spinodal decomposition (SD), to the case of enhanced atomic mobility at the GB. As the result, a microstructure resembling DP was obtained and the whole the process was called discontinuous SD. However, the main differences between a classical DP reaction and discontinuous SD is that in DP, one of the lamellae must be the precipitate phase, whose composition is significantly different from that of the matrix. In the discontinuous SD, the lamellae are created due to a composition wave of relatively large amplitude, but simultaneously coherent interfaces exist between the solute-rich and solute-depleted lamellae.

Amirouche and Plapp [3,4] developed the PF model for the DP reaction by modifying an existing solution for the solidification. They used the so-called the multi-phase-field approach, in which each phase (α_ο_, α, β) was described by one phase field and applied it for only isothermal growth in a binary alloy. The simulation showed that the bulk diffusion was important for the formation of solute-rich precipitates and initiation of the DP reaction, and that the steady-state growth rates depended generally on both bulk and surface diffusivities, as well as on the grain boundary mobility. One should note, that all the considerations of Amirouche and Plapp [4] referred to the solute-rich lamellae as being, in all the simulations, much thicker than the solute-depleted lamellae. Moreover, the values of the precipitate growth velocity obtained from their simulations did not agree with the theoretical predictions. As Amirouche and Plapp [4] admitted, such a discrepancy was due to the fact that the local equilibrium hypothesis used in their sharp-interface model was not valid for diffusive interphase boundaries in the presence of strong surface diffusion.

Quite recently, Duong et al. [5] applied the so-called multiscale modelling for the DP in U-Nb alloy, which included first-principle calculations and CALPHAD to assess the self-consistent thermodynamic description of the U-Nb system. Then, atomic mobility and diffusivity was determined again using CALPHAD via the DICTRA package. Finally, they used the phase-field interface dissipation model developed by Zhang, Steinbach, and Plapp [6,7], which does not require local equilibrium conditions at phase interfaces and the degree to which this quasi-equilibrium condition is enforced can be governed by a so-called permeability parameter that controls how easy or difficult is for solutes to cross phase interfaces. It was especially useful because Duong et al. [5] considered formation of the metastable γ′ phase during the reaction γ → α + γ′, with an intermediate composition differing from that of the stable γ_2_, which forms during subsequent discontinuous coarsening. Their results showed further progress and the important role of interfacial strain in the stabilization of the DP product, and also that highly anisotropic boundary diffusion can stabilize the DP front.

One of the most popular computational alternatives to the PF method is a cellular automata (CA) method, which is successfully used to model solid-state phase transformations. There are a number of works [8,9,10,11,12] presenting capabilities of models based on CA, predominantly for meso-scale simulations. However, it is worth pointing out that a number of CA models were recently developed to model physical phenomena at the micro-scale [13,14], and even down to nano-scale to simulate diffusion in nanocrystalline materials. For the latter case, Zhao et al. [13] presented a numerical scheme considering both grain growth and nitrogen diffusion in the nanocrystalline structure of iron. On the other hand, Shen et al. [14] applied CA model, which depicted the interface evolution inside the cementite plus ferrite lamellar microstructures during the reaustenitisation of pearlite steel according to the overall scheme: (α + β → γ). In a consequence, this resembles the discontinuous dissolution, reverse reaction to the DP, which can be written as: α + β → α_ο_. A crucial aspect of this work is a very fine grid spacing set as 1/50 of the thickness of a cementite lamella, which finally gives the cell size of the cellular automaton at the level of 10 nm. This is promising for the successful building of a physically-based CA model dedicated to the discontinuous precipitation, which has to consider the set of individual α and β lamellae.

The purpose of this work is to present the fundamentals of cellular automata modelling of the DP reaction. We will focus on presenting a general application concept of the CA model for the DP, assumptions for the reaction front discretisation, and necessary simplifications, which are required for the first approximation of DP reaction modelling using the CA approach. Furthermore, a scheme of a digital material representation for the initial microstructure in simulations will be carefully depicted. The simulation settings with crucial parameters of the model will be presented. Finally, the capabilities of the CA model dedicated to the DP reaction will be demonstrated and discussed.

## 2. Model Concept

### 2.1. General Idea of the CA Model for DP Reaction

**Figure 1:**
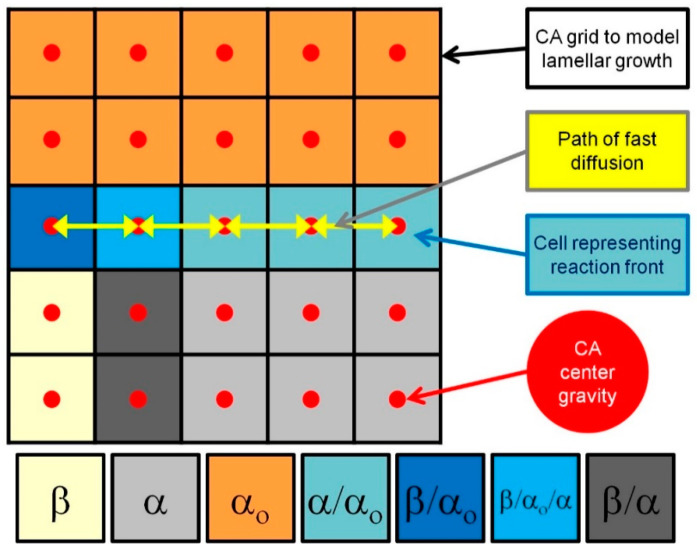
Grid scheme presenting CA grid to model lamellar growth and solute fast diffusion.

During discontinuous precipitation, two individual growing phase processes into supersaturated solid solution α_o_ can be distinguished. The first one is associated with a solute-rich β phase lamella, and the other is associated with a solute-depleted α phase lamella, with the same crystal structure as the initial parent α_o_ phase. On this basis, one can distinguish at least two types of interfacial boundaries β/α_o_ and α/α_o_, which separate growing new phases from the parent phase. Discontinuous precipitates nucleating at grain boundaries and during growth a cellular structure, i.e., alternating lamellae of β and α phases are formed, between which a β/α interfacial boundary exists. These three phases come into a contact at a single point, the so-called triple point, which is defined as the β/α_o_/α interfacial boundary. All phases and types of interfacial boundaries that occur during the DP reaction are shown in Figure 1 via a grid diagram of the cellular automata. In the CA model computation process, each cell represents only one phase or interfacial boundary state.

The CA method is a well-known numerical technique, which is widely used in the modelling of phase transformations in solid state [8,9,10,11,12,13,14]. The classical definition of the CA method can be found in Chopard’s work [15], and its more expanded description consists of five basic concepts: the space of cellular automata (Ω), i.e., a grid of cells that serves for discretisation of the modelling area; a finite set of states (Y), by which individual cells are identified in the computational domain; a function that assigns the appropriate state to each cell (A); cell neighbourhoods (N), which are used predominately in determining the new state of a cellular automaton; and transition rules (P), which control changes in cell states. In mathematical form, this can be represented as follows:

(1) CA=〈Ω,Y,A,N,P〉

**Figure 2:**
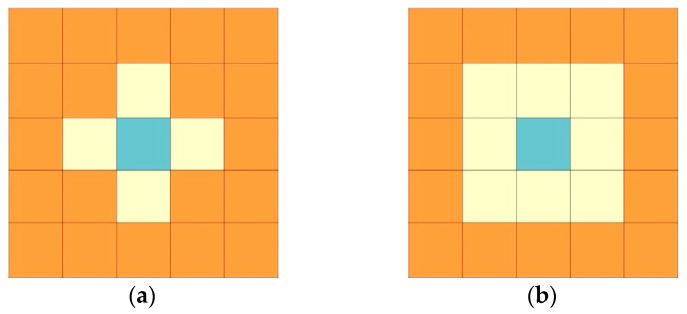
Types of neighbourhood in the cellular automata space: (**a**) von Neumann (4 nearest cells); (**b**) Moore (8 nearest cells).

Two types of cell neighbourhood, most commonly used in the CA model, are presented in Figure 2. The first one is a von Neumann neighbourhood, which is used both in defining the growth conditions of a new phase and in solving the diffusion equation. The second is Moore’s neighbourhood, which is used in setting initial conditions and in the growth model. The evolution of the system is determined by transition rules, which are called in each time step synchronously to define the current internal variable describing the state of each cell, based on the previous states of its neighbours and the cell itself. Generally, the transition rules are precisely defined based on additional sub-models.

### 2.2. Main Assumptions

In the present CA model, the modelling space is discretised onto a two-dimensional regular, equally spaced square grid (Figure 1), which constitutes a digital material representation (DMR) of the microstructure fragment. Due to the attribute of the CA method, it is possible to implicitly track the position of the reaction front and to introduce new phases and grains into a virtual microstructure built as a DMR. In many works [10,11,12], the approach of integrating the CA method with the finite difference method (FD) is used, resulting in a solute diffusion calculation in the modelling space based on the solution of Fick’s 2nd law equation. An alternative way to solve the diffusion equation in the paradigm of the cellular automata is demonstrated in the work of Chopard and Droz [16]. However, another calculation approach can be used that considers the cellular automaton centre as an integration point in which modelling parameters, e.g., solute concentration, can be determined according to analytical methods, e.g., based on Cahn’s equation [17], as is later presented in this paper. A scheme of the CA grid with marked centres, which enables simultaneous modelling of the new phase precipitates growth and the solute diffusion, is shown in Figure 1.

In order to properly describe the phenomena that occur during discontinuous precipitation, the cells in the CA model are characterized by four state variables:

(1) the phase state, which defines that a cell represents α_o_, α, β phases or α/α_o_, β/α_o_, β/α_o_/α interfaces, and immobile β/α interphase boundary;
(2) the solute concentration variable, which includes an average solute concentration in a cellular automaton;
(3) the α fraction variable, which quantifies the degree of α_o_ → α transformation at the interface cell;
(4) the β fraction variable, which quantifies the degree of α_o_ → β transformation at the interface cell.

Furthermore, the following assumptions need to be taken in the implementation of the computational algorithm with the CA model for the discontinuous precipitation:

- A sharp interface (SI) model is applied in cellular automata representing interfacial boundaries, i.e., reaction fronts. In the sharp interface formulation, the interphase boundary is treated as a singularity at which a step-change in material properties is occurring;
- Reaction front tracking was established to be implicit in cellular automata, representing interfacial boundaries, due to the excessively low resolution (low density) of the CA grid;
- In the growth calculation, the interfacial boundary at the β/α_o_/α triple point is treated as an α/α_o_ reaction front where the transformation follows the α_o_ → α pattern;
- The effect of the initial microstructure on further DP is accounted for in the CA grid by a scheme of digital material representation;
- Direct modelling of solute diffusion using the differential equation of Fick’s 2nd law is omitted, and an analytical solution of the diffusion equation for the DP reaction provided by Cahn [17] is adopted instead;
- The nucleation process of the growing phase is neglected because only steady-state growth during the DP reaction is considered in the model;
- Establishment of uniform temperature field throughout the cellular automata space, because of the microscopic scale of the modelling space;
- Any temperature effects from the phase transformation, e.g., recalescence effect, are not accounted for in the modelling. The isothermal process is considered in the simulations in the ideal thermal conditions.

### 2.3. Sharp Interface Model

**Figure 3:**
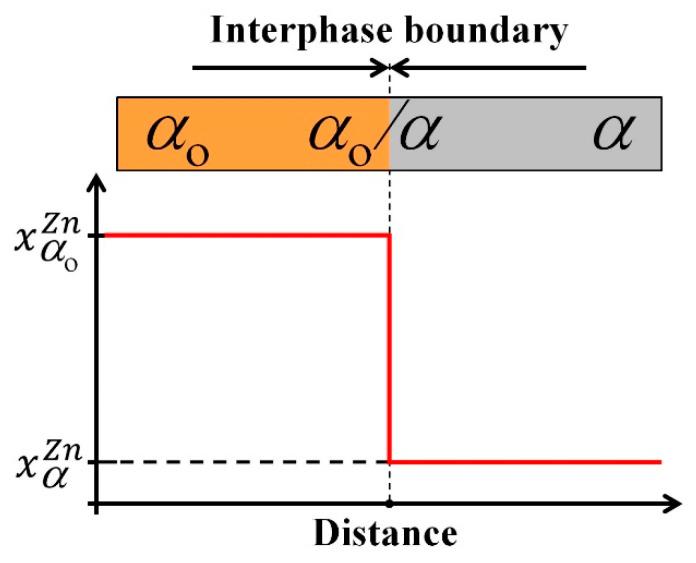
Sharp interface concept used to discretise the chemical discontinuity across the reaction front.

One of the crucial assumptions made in building the CA model for the DP reaction was the approximation of RF using the sharp interface concept. This approach was verified by analysing the experimental results. Microanalytical studies of the DP reaction confirmed that a chemical discontinuity occurs across the RF in a direction perpendicular to the position of the original boundary [18,19,20,21]. Figure 3 is a schematic illustration of the sharp (discontinuous) change of the solute content across the RF (α_o_ → α transition), which was an assumed discretisation of the reaction front. It means that the nano-area of the interface is treated as an infinitesimally thin layer, with a practical zero width, in a numerical implementation of the algorithm. In effect, a sharp skip of the physical properties of grains and phases in the modelled area of the RF was established (see Figure 3).

### 2.4. Digital Material Representation

**Figure 4:**
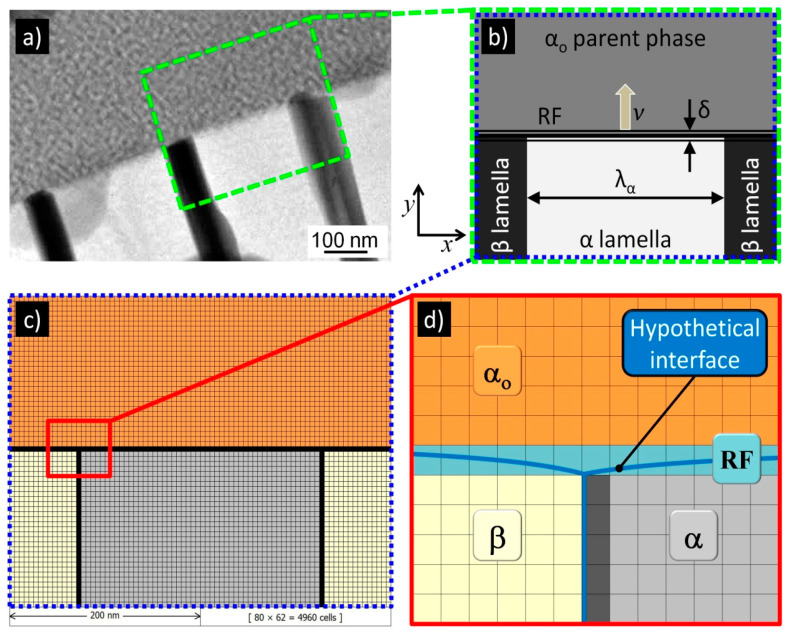
Scheme showing the concept of digital material representation based on digitalisation of the metallographic image (**a**) through the reproduced arrangement of an individual cell for the DP reaction (**b**) and its conversion into the cellular automata grid (**c**). Zoomed fragment of the CA grid (**d**) presents the hypothetical interface (RF) and, in effect, the implicit character of its tracking in the CA model.

In order to reproduce the initial microstructure for numerical simulations of the DP reaction, a DMR approach proposed by Opara et al. [22] was adopted in the current issue. The solution is based on a dedicated algorithm for the conversion of a raster image of the investigated microstructure into the digital material representation using the CA method. The proposed approach consists of the following steps:

- Manual redrawing of the selected area from microstructure, presented in Figure 4a, as a raster image with schematic cellular structure (with two β phase lamellae, one α phase lamella, and one α_o_ phase grain), as is demonstrated in Figure 4b, maintaining the proportions between lamellae;
- Reading the raster image (Figure 4b) with four assigned colours to each phase and grain boundaries, i.e., grey to α_o_ phase, light grey to α phase, graphite to β phase, and black to RF, into a computer program based on the dedicated CA algorithm that is able to recognize and properly interpret DP structure, keeping its real dimensions read from the marker scale size depicted in Figure 4a;
- Conversion of the interpreted raster image with DP structure into the CA grid (Figure 4c) with the assumed discretisation precision of the physical space, that determines the size of the cellular automaton and consequently the magnitude of the CA grid;
- Initialization of internal variables of cellular automata (i.e., phase state, solute concentration, and fraction of the considered phase) according to the colour of the specified phase or grain boundary. Details of the initial microstructure setting with specific properties are presented in Section 3.

An example of the DMR, with a slice of the initial microstructure for the numerical simulations, is shown in Figure 4c. In turn, Figure 4d presents a zoomed-in section of the DMR that accurately shows the cellular automata discretising the reaction front and the grains belonging to the three different phases. The characteristic feature in the adopted solution in the CA method is the implicit tracking of the RF position in cells representing interfacial boundaries, the hypothetical course of which is outlined in Figure 4d.

### 2.5. Mathematical Modelling of DP

Hillert [23], followed by Klinger et al. [24], proposed an analytical approach to model the discontinuous precipitation reaction with the individual treatment of the growth of each newly emerging α and β phase. Since the growth process of each phase occurs according to a different mechanism, Klinger et al. [24] introduced applicable equations to describe the migration velocity for two types of interfacial boundaries (β/α_o_ and α/α_o_). It should be noted that each of the adopted equations is based on the fundamental assumption that the motion of the interfacial boundary is controlled by the local equilibrium of forces acting in the interface [25], which is expressed by the following general relation:

(2) v=M(G−Γ)

where v denotes the linear growth rate of the reaction front, *M* is the local mobility of the interfacial boundary, *G* is the chemical driving force of the phase transformation (Gibbs free energy change), and *Γ* is the local capillary force, representing the Gibbs-Thomson effect associated with the curvature of the interfacial boundary. The Formula (2) is equivalent to the relationship presented in Christian’s work [26]. This equation describes the linear relationship between the migration velocity of the interfacial boundary and the total chemical driving force of the transformation at non-equilibrium thermodynamic conditions close to the equilibrium state. The crucial point in the proposed DP reaction modelling approach is the fulfilment of several basic conditions, i.e., the preservation of mass balance at the interface between grain boundaries and reaction fronts (β/α_o_ and α/α_o_) set the identical growth rates in adjacent α and β lamellae so that the system reaches the steady-state, which is considered in this work.

Consequently, in the first approximation of DP modelling using the CA method, the migration velocity of the interfacial boundary was assumed to be constant under certain conditions and for a certain time in each cellular automaton representing the RF. Under this assumption, it is easy to determine the solute concentration profile (*x*) in the α lamella that arises in the *z*-axis parallel to the moving α/α_o_ reaction front, based on the Cahn equation [17] in the following form:

(3) $$x\left(z\right)=xₒ-\left(xₒ-x_{\alpha /\beta }\right)\frac{\cosh\left[\left(z-0.5\right)\sqrt{C}\right]}{\cosh\left(0.5\sqrt{C}\right)}$$

in which the parameter *C* is expressed by the formula:

(4) $$C=\frac{v\lambda_{\alpha }^{2}}{s\delta D_{b}}$$

where *x*_o_ is the average concentration of the solute in the alloy, *x*_α/β_ is the concentration of the solute in the α-phase lamella at the interfacial boundary with the β-phase lamella, *λ*_α_ denotes the width of the lamella α, *s* is the segregation factor, *δ* is the width of the grain boundary (RF), and *D*_b_ is the grain boundary diffusion coefficient of the solute.

A similar approach for determining the solute concentration profile using an analytical equation was applied in the context of modelling the carbon concentration profile at the interfacial boundary front during the austenite to ferrite phase transformation by Bos and Sietsma [8,27]. However, one should note that the model of Bos and Sietsma [27] was used to determine a solute profile, which is perpendicularly directed to the interface during simulation of the volume diffusion, whereas Equation (3) is used to simulate a solute profile along of the interface during grain boundary diffusion process. Nevertheless, both models allow for performing computationally efficient simulations within a reasonable time.

### 2.6. CA Algorithm

The transition rules in CA models dedicated to the microstructure evolution of polycrystalline systems are usually based on an equation describing the grain growth length of the arising phase in cellular automata, representing the interfacial boundary (*i*—interface) [8]. The growth length parameter ($l_{\mathrm{cell}}^{i}$) is calculated at each time step (Δt) by integrating after time (*t*) the migration velocity of the interface (v), according to an explicit Euler scheme:

(5) $$l_{\mathrm{cell}}^{i}\left(t+\Delta t\right)=l_{\mathrm{cell}}^{i}\left(t\right)+v\Delta t$$

Further, according to this approach, a volume fraction of the transformation is determined in the CA computational algorithm, only in frontal cellular automata:

(6) $$F_{\mathrm{cell}}^{i}=\frac{l_{\mathrm{cell}}^{i}}{L_{\mathrm{CA}}}$$

where $L_{\mathrm{CA}}$ is the size of the cellular automaton, also known as the side width of CA, which can also be interpreted as the constant distance between the centres of gravity of the two neighbouring cellular automatons contacting each other by sides (the von Neumann neighbourhood of Figure 2a). Based on Equation (6), the following transition rules (Equations (7)–(9)) were formulated to determine the growth of the newly formed phase with the change in the concentration of the solute in the α-phase lamella using relation (3):

(7) $$Y^{i}\left(t+\Delta t\right)=\{\begin{array}{c} \alpha \;\Leftrightarrow \;F_{\mathrm{cell}}^{i}\geq 1\;\wedge \;Y^{i}\left(t\right)=\mathrm{RF}\\ Y^{i}\left(t\right)\;\;\;\;\;\;\;\;\;\;\;\;\;\;\;\;\;\;\;\;\;\;\;\;\;\;\;\;\;\;\;\;\;\;\;\;\;\;\;\;\;\; \end{array}$$

(8) $$x^{i}\left(t+\Delta t\right)=\{\begin{array}{l} \mathrm{eq}\left(3\right)\;\Leftrightarrow \;Y^{i}\left(t+\Delta t\right)=\alpha \\ x^{i}\left(t\right) \end{array}$$

(9) $$Y^{j}\left(t+\Delta t\right)=\{\begin{array}{l} \mathrm{RF}\;\Leftrightarrow \;Y^{j}\left(t\right)=\alpha_{o}\;\wedge \;Y^{i}\left(t+\Delta t\right)=\alpha \\ Y^{j}\left(t\right) \end{array}\;\;\;\;\;\;j\in N_{N}\left(i\right)$$

where $Y^{i}$ is the state of the cellular automaton with interphase boundary (*i*) in which the growth calculation of the migrating RF is performed, $Y^{j}$ is the state of a cellular automaton from the nearest neighbourhood of frontal cells, $N_{N}\left(i\right)$ is the von Neumann neighbourhood of the cellular automaton representing the interphase boundary, and $x^{i}$ is an internal variable that specifies the average concentration of a solute in a cellular automaton with an interphase boundary. For the growth of the β-phase lamella, the transition rules (10)–(12), are as follows:

(10) $$Y^{i}\left(t+\Delta t\right)=\{\begin{array}{c} \beta \;\Leftrightarrow \;F_{\mathrm{cell}}^{i}\geq 1\;\wedge \;Y^{i}\left(t\right)=\mathrm{RF}\\ Y^{i}\left(t\right)\;\;\;\;\;\;\;\;\;\;\;\;\;\;\;\;\;\;\;\;\;\;\;\;\;\;\;\;\;\;\;\;\;\;\;\;\;\;\;\;\;\; \end{array}$$

(11) $$x^{i}\left(t+\Delta t\right)=\{\begin{array}{l} x_{\beta }\;\Leftrightarrow \;Y^{i}\left(t+\Delta t\right)=\beta \\ x^{i}\left(t\right) \end{array}$$

(12) $$Y^{j}\left(t+\Delta t\right)=\{\begin{array}{l} \mathrm{RF}\;\Leftrightarrow \;Y^{j}\left(t\right)=\alpha_{o}\;\wedge \;Y^{i}\left(t+\Delta t\right)=\beta \\ Y^{j}\left(t\right) \end{array}\;\;\;\;\;\;j\in N_{N}\left(i\right)$$

where *x*_β_ is the constant concentration of the solute in the β-phase lamella, which is taken from the phase equilibrium diagram of the chemical system under consideration at a given temperature.

The set of Equations (7)–(12), with cellular automaton transition rules that are invoked at each computational step, i.e., after the change of discrete segment of time, can be described as follows. When the volume fraction of transformation ($F_{\mathrm{cell}}^{i}$) in a frontal cell (in the RF state) exceeds the value of 1, and thus its growth length ($l_{\mathrm{cell}}^{i}$) reaches the value of the cellular automata size (*L*_CA_), the cell changes its so-called transition state (RF state) into the phase state of growing lamella α or β, depending on the transition rule (7) or (10). Immediately after this operation, a new value is assigned to the variable that specifies the average concentration of the solute in the cellular automaton, which transformed into the α or β phases, based on Equation (3), or a constant value of *x*_β_, depending on the (8) or (11) transition rule. Thereafter, the parent phase cells (α_o_) from the closest neighbourhood (von Neumann neighbourhood) of the cellular automaton, on which the new phase was formed, are transformed into frontal cells (RF) according to (9) or (12) rules, and their growth length ($l_{\mathrm{cell}}^{i}$) in subsequent steps is calculated according to Equation (5).

The essence of solving the problem of modelling the phase transformation of discontinuous precipitation by the method of cellular automata is to apply the mathematical Equations (3)–(12) to calculate the growth kinetics and the concentration of the solute in the α phase lamella. The results of computing these parameters are assigned to internal variables from the state space of the cellular automaton. Afterward, based on the values of these variables in the considered cell and its vicinity, i.e., the nearest neighbourhood with an assumed range, the state transition rules that determine the evolution of the system are defined. The presented description of the two-dimensional discrete model, based on the cellular automata method, was implemented in the C++ language using an object-oriented programming (OOP) technique in the form of a computer program with graphical user interface.

### 2.7. Time Step Definition and Speed Up of Computations

In order to ensure the stability of the numerical calculation, during the simulation of the discontinuous precipitation phase transformation, the time step was dynamically adjusted according to the velocity of the migrating reaction front, the boundary diffusion coefficient of the solute, and the side width of the cellular automata, according to the following equation:

(13) $$\Delta t=0.9\cdot \min\left\{\frac{L_{\mathrm{CA}}}{v},\frac{{\left(L_{\mathrm{CA}}\right)}^{2}}{4D_{b}}\right\}$$

Thus, any change in chemical composition and structure in the grain volume and at migrating boundaries of discontinuous precipitates is directly reflected in the kinetics of phase transformation during numerical simulations. Additionally, a proprietary solution was introduced in the main loop of the computational algorithm to speed up the computation by estimating after which time the next increase in the newly formed phase would occur and, based on this, optimally increasing the phase increment in the transition cells and the local time step. As a result, bearing in mind that the growth was concerned at the nanoscale, a significant acceleration of the calculations was obtained (from tens of hours to several seconds), while retaining the original time step value information resulting from the application of Equation (13), as well as the hypothetical number of function calls with CA model transition rules, reaching the value of several hundred billion CA steps (CAs). Such an approach is only possible if the growth rate of the migrating reaction front is assumed to be constant and is identical across all frontal cellular automata from the entire computational domain, which was the case in the proposed approach of modelling DP reactions with CA.

Since the computation of microstructure evolution in the modelling system is related only to cellular automata representing interfacial boundaries, the concept of frontal cellular automata (FCA), proposed by Svyetlichnyy [28], was used in the implementation of the CA model. The solution relies on selecting from the entire modelling space, the cellular automata in the so-called transition state, where phase growth calculations at the RF are performed. Hence, the name frontal cellular automata, in which the direction of information transfer is the opposite to the classical approach, i.e., the cell under consideration sends out information about its state to cells in the nearest vicinity, rather than receiving this data. As a result, significant acceleration of computation time is obtained, which is related to the fact that large areas of cellular automata belonging to the interior of the grains, which are not directly involved in the operations of checking and executing the growth transition rules of new phases, are omitted.

## 3. Simulation Setting

Numerical simulations of the steady-state growth of discontinuous precipitates were performed at the example of Al-22 at.% Zn alloy, which was extensively reported in the literature in terms of DP reaction, including studies of solute concentration profiles across the α-lamella and across the reaction front, using analytical electron microscopy (AEM) (FEI, Hillsboro, OR, USA) [20], and in situ observations by transmission electron microscopy (TEM) (FEI, Hillsboro, OR, USA) [29]. The initial conditions of the simulation were established based on experimental data and numerical tests presented in [30]. Hence, it was assumed that the simulations of the DP reaction would be performed for the isothermal conditions at 400 K (~127 °C). Based on the phase equilibrium diagram of the Al-Zn system [31], the relevant Zn concentrations in the α and β phases were: *x*_α/β_ = 4.4 at.% Zn and *x*_β_ = 99 at.% Zn, respectively. In order to smoothly control the simulation process of discontinuous precipitates growth, the values of the parameter *C*, as a function of time, were introduced in the code of the computer program with the implemented CA model for the DP reaction, using the data submitted in [30]. This made it possible to run numerical simulations for the assumed constant RF migration velocity over a certain observation time, taking into account the go-and-stop motion during movement of the interface.

**Figure 5:**
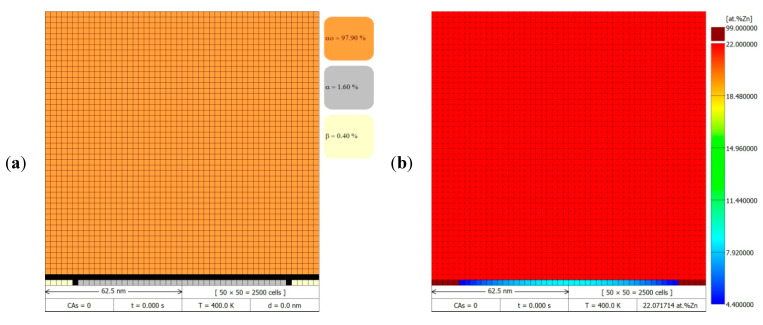

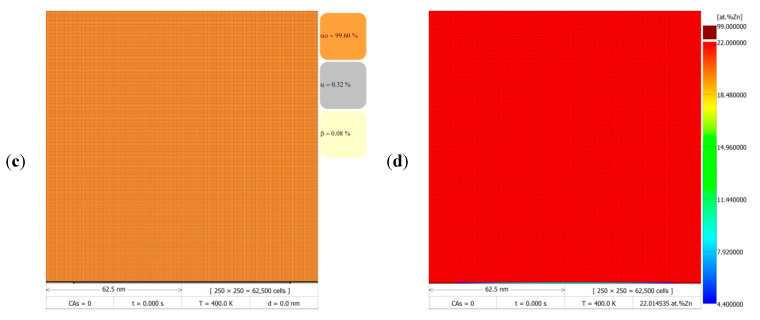
Initial conditions in the digital material representation of the microstructure fragment (125 × 125 nm^2^), for two cases of discretisation with a CA grid of size: (**a**,**b**) 50 × 50 = 2500 cells, and (**c**,**d**) 250 × 250 = 62,500 cells, where left column presents the current phases and right column depicts maps with zinc redistribution.

The initial microstructure used in the numerical simulations of the DP reaction was generated according to the DMR concept on a two-dimensional grid of cellular automata, in the shape of squares, which was presented previously in Figure 4c with a schematic section of the microstructure. It was assumed that in the modelling space only a set containing the single α phase lamellae with two neighbouring β phase lamellae and the surrounding α_o_ parent phase was considered. The growth of the precipitates occurs in the *y*-axis according to the Cartesian coordinate system. The discontinuous precipitate lamellae are found to be at an early stage of growth (just after nucleation), so their height is small and occupies the size of only one cellular automaton (*L*_CA_). Taking the width of the α lamella as equal to 100 nm (see Table 1 in [30]) and the width of lamella β as 8 times smaller, the resulting width of the modelling area was 125 nm. The modelling area was assumed to be square-shaped (Figure 5) and therefore its height was also 125 nm.

The modelling system determines the adoption of reflective (mirror) boundary conditions at the edges of the CA grid to ensure the stability of the solution and to avoid different phases coming into contact on opposite sides of the CA grid edges, which would lead to undesirable modelling effects. Some phases present in the modelling space were assigned with a homogeneous solute concentration, as shown in Figure 5b,d. It was assumed that the Zn-content in the α_o_ parent phase was constant and equal to the average zinc concentration (*x*_o_) in the Al-Zn alloy. Similarly, in the β-phase lamellae the zinc concentration did not change during the simulation and was equal to *x*_β_. In turn, the initial zinc concentration in the α phase lamella (*x*_α_) was determined using Equation (3) and based on the preliminary conditions for each simulation. In the initial microstructure in the FCA representing the interfacial boundaries, the internal variables with volume fractions of the individual phases were assumed to be established on the basis of adopting 100% of a given phase from the nearest Moore neighbourhood in which that phase globally had the largest surface contribution. According to this relationship, the zinc concentration in the transition cells was specified following a mass balance, from which results that in the immobile β/α interfacial boundary the zinc concentration was the same as that in the α-phase lamella (*x*_α_). On the other hand, in the cells in contact with RF (β/α_o_, α/α_o_, β/α_o_/α), the zinc concentration takes the value *x*_o_ as in the α_o_ parent phase grain. In order to facilitate the interpretation of the results in 2D maps with the redistribution of the solute, the zinc concentration in the β-phase lamellae was set to be represented by a single colour, maroon, due to the fact that the zinc concentration in the β-phase does not change during the simulation and is very high (~99 at.% Zn). The zinc concentration in the other phases was presented in a range from blue (lowest Zn concentration) to red (highest Zn concentration). The value of the zinc diffusion coefficient in the Al-Zn alloy was determined based on the Arrhenius equation and the diffusion parameters (after conversion to SI units: $D_{b0}^{Zn}$ = 3.2 × 10^−6^ m^2^/s, $Q_{b}^{Zn}$ = 56 103 J/mol) presented in Hässner’s work [32].

Simulations of the DP transformation were performed for different RF migration velocities (v = 10 nm/s, 20 nm/s), according to the data taken from Table 1 in [30]. The results obtained from the simulations were used to initially verify the assumptions made in the developed CA model for the DP transformation. Simulations were carried out for two discretisation cases on a CA grid to check how the size of the cellular automaton will affect the computational accuracy and execution time. Depending on the adopted side width of the cellular automaton of $L_{\mathrm{CA}}$ = 2.5 nm or $L_{\mathrm{CA}}$ = 0.5 nm, the size of the CA space and, thus, the discretisation density changed, as shown in the DMR images with the visible cell grid in Figure 5a,c. In the first case, the physical region of modelling of 125 × 125 nm^2^ was discretised into a CA grid of size 50 × 50, i.e., 2500 cells, and in the second case the CA grid size was 250 × 250, i.e., 62,500 cells.

## 4. Results and Discussion

**Figure 6:**
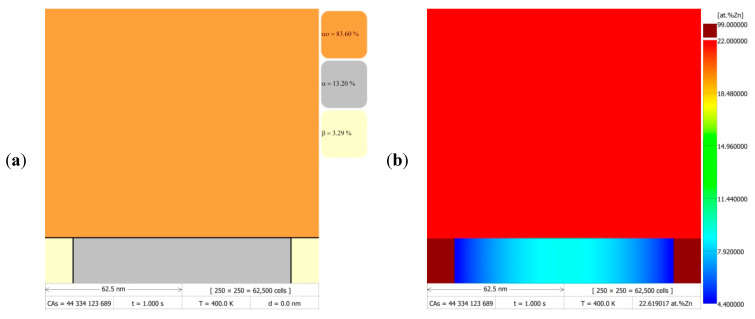

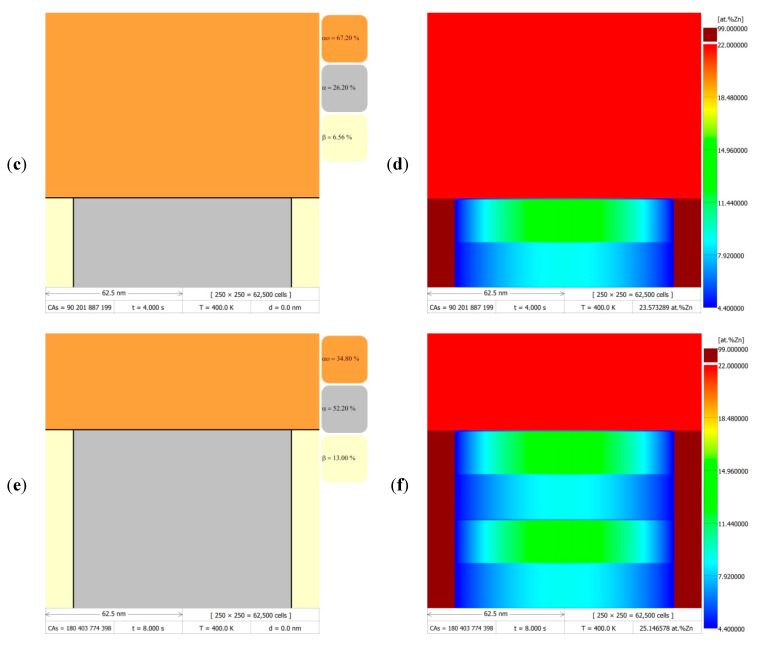
Simulation results in the form of DMR images (250 × 250 cell CA grid) with visible phases (left column) and maps with zinc redistribution (right column), which show the microstructure evolution and chemical composition changes during the DP reaction at 400 K (~127 °C), for a fixed RF migration velocity, v = 20 nm/s, after time: 1 s (**a**,**b**); 4 s (**c**,**d**); and 8 s (**e**,**f**).

Figure 6 presents the results of the numerical simulations of phase transformations at the boundaries of discontinuous precipitates migrating at a constant velocity of v = 20 nm/s at 400 K (~127 °C) for 8 s. Figure 6a,c,e, show the successive stages of microstructure evolution in the form of DMR images (grid size 250 × 250 cells) with visible phases and grain boundaries after 1, 4, and 8 s, respectively. The corresponding zinc redistribution maps in Figure 6b,d,f, show the changes in chemical composition occurring during the discontinuous precipitation reaction. Due to the compilation of the DMR images in Figure 6, which were generated using a computer program with the implemented CA model, it is possible to directly observe the changes in chemical composition in the α lamella with the migration of discontinuous precipitates and stopping this process for a certain period of time, the so-called go-and-stop motion cycle. The presented simulation results demonstrated two complete cycles with RF displacement and stop, during which the zinc concentration relaxed along the α/α_o_ boundary at the fourth and eighth second of the DP transformation. During the simulation of one cycle, the zinc concentration profile across the α phase lamella, parallel to the migrating reaction front, changed stepwise between specific time intervals, depending on the adopted value of the *C* parameter.

**Figure 7:**
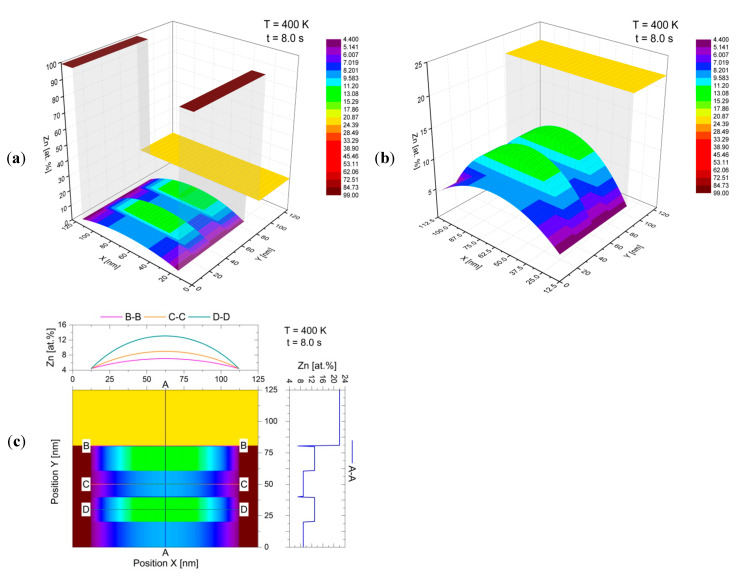
Simulation results after 8 s of DP reaction at 400 K (~127 °C) for a fixed RF migration velocity v = 20 nm/s, in the form of: three-dimensional surface plots with the whole modelling area (**a**) and with the α lamella only (**b**); map with line markings of zinc concentration at several locations for the α lamella (**c**).

This effect is even more clearly demonstrated by the three-dimensional surface plots in Figure 7a,b, as well as by the map with the linear zinc concentration determination (analogous to those from the AEM studies), made parallel and perpendicular to the migrating reaction front, which is presented in Figure 7c. On the basis of the data presented in Figure 5 and Figure 6, the simulation results can be considered to be in qualitative agreement with the observations of discontinuous precipitation by means of [19,20,21,29,30], while maintaining the appropriate proportions that resulted from the adopted simplifications, namely considering a schematic modelling region consisting of a microstructure slice in which only two β-phase lamellae and one α-phase lamella grow parallel to the *y*-axis (according to the Cartesian coordinate system). This provides a positive verification of the assumptions made in building a two-dimensional discrete model, based on the CA method, for the simulation of solid-state phase transformations at migrating boundaries of discontinuous precipitates.

**Figure 8:**
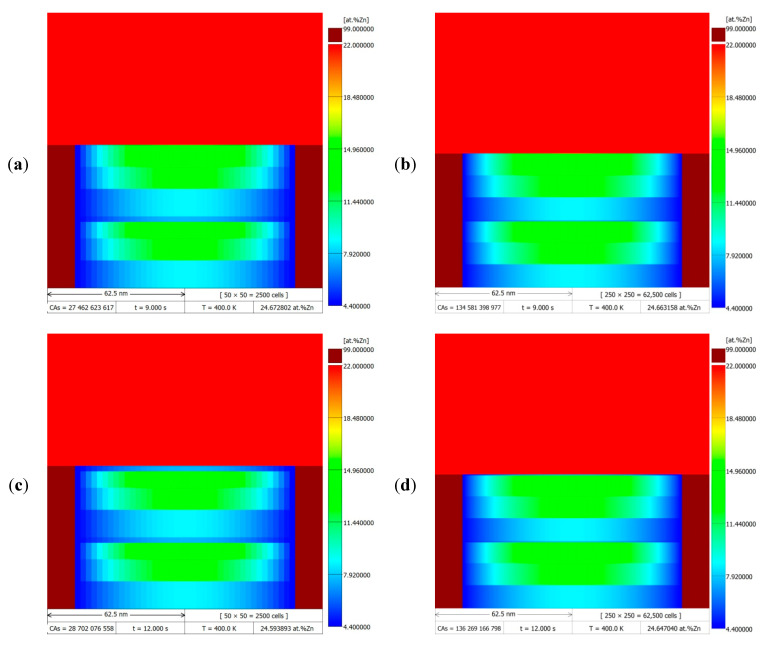
DMR images with the zinc redistribution maps after simulation of DP transformation with migrating RFs at constant velocity v = 10 nm/s at 400 K (~127 °C), after 9 and 12 s, for different cases of CA space discretisation with sizes: 50 × 50 cells with $L_{\mathrm{CA}}$= 2.5 nm (**a**,**c**); 250 × 250 cells with $L_{\mathrm{CA}}$= 0.5 nm (**b**,**d**).

Figure 8 shows the simulation results of the DP transformation for a migrating RF with constant velocity v = 10 nm/s at 400 K (~127 °C), after a time of 9 and 12 s. Simulations were performed for two CA space discretisation cases (50 × 50 with $L_{\mathrm{CA}}$ = 2.5 nm and 250 × 250 with $L_{\mathrm{CA}}$ = 0.5 nm), the results of which are presented as DMR images behind the zinc redistribution maps. Comparing the results in the left column with those on the right, there was a noticeable difference in the resolution of the presented zinc concentration values in the α-phase lamella as a colour gradient, which is a direct result of the discretisation density of the modelling area. In the first case with a lower discretisation density, jumps between Zn concentration values were visible, making it easier to see changes in the zinc concentration profile, e.g., at the stage of relaxation of the Zn concentration during the stopping period of the migrating RF. On the other hand, for the case of discretisation with a higher degree of density, smoother changes in the values of zinc concentration in the microstructure could be observed.

The density degree of the discretisation of the modelling area also affected the size of the time step through the CA cell size used in its determination according to Equation (13), and, thus, the number of function calls with CA transition rules in the main computation loop, and consequently the actual time of the numerical simulations performed. The higher the discretisation density, the smaller the time step and thus the higher the number of CA transition rule calls and the longer the computation time, which is confirmed in Table 1 by the collation of the simulation results. Furthermore, the compilation of data in Table 1 is significant in that it gives an outlook of the computational complexity when formulating a second approximation to model the DP reaction using the CA method including solute diffusion calculations according to Fick’s 2nd law with a numerical solution, e.g., using the FD method, as the much larger simulation times are to be expected. Then, the diffusion calculations will have to be performed on the whole CA grid at each transition step with a very small time step, and the solutions adopted in the presented CA model for accelerating the calculations, such as local time step increase and global estimation of the number of CA transitions, would not be applicable.

## 5. Conclusions

This paper presents the description of a two-dimensional discrete model based on the cellular automata method, which was used for the modelling of solid-state phase transformations at migrating boundaries of discontinuous precipitates. In the proposed CA model, cell states, internal variables, equations, and transition rules were defined to predict the manner of mass transport during discontinuous precipitation and to relate changes in microstructure during discontinuous precipitation with the changes in chemical composition. In the CA model, the concept of digital material representation was introduced, which allowed schematic images of the microstructure to be used as starting structures in the modelling of the DP reaction. As a result, it was possible to initiate the simulation under conditions as close as possible to the experimental observations.

The preliminary assumptions adopted in the proposed CA model for the DP reaction were verified by numerical simulations of the growth of discontinuous precipitates at a steady-state on the example of Al-22 at.% Zn alloy. The outcomes achieved from the CA simulations were presented in a different form than that most commonly used previously (single concentration profiles), namely as the 2D maps showing changes in Zn content accompanying the successive stages of the growth of the discontinuous precipitates. By discretising the modelling area in two spatial dimensions, the results were recorded in the form of matrices on which linear analyses were carried out with quantitative determinations of zinc concentration (Figure 7c), performed parallel and perpendicular to the migrating reaction front (analogous to studies using AEM). In effect, a qualitative and quantitative comparison of simulation results with experimental outcomes was possible. The model used for the description of the solute diffusion along the reaction front allowed two-dimensional systems at the nano-scale to be treated within a reasonable simulation time. The obtained results indicate that the developed CA model is able to realistically simulate the DP reaction, which is confirmed by the visualisation of migrating RFs together with the associated chemical composition changes in the microstructure.

## Figures and Tables

**Table 1:** Summary of results informing computational complexity of the CA model, depending on the discretisation density of the modelling area, after simulations of the DP transformation under RF migration conditions with constant velocity v = 10 nm/s at 400 K (~127 °C).

Modelling Area	Magnitude of the Space of Cellular Automata	Size of the Cellular Automaton	Time Step	Estimated Number of CA Steps
125 × 125 nm^2^	50 × 50 cells	2.5 nm	2.25 × 10^−10^ s	28,702,076,558
	250 × 250 cells	0.5 nm	4.50 × 10^−11^ s	136,269,166,798

## Data Availability

The data presented in this study are available on request from the corresponding author.
